# Disability and Accommodation Use in US Bachelor of Science in Nursing Programs

**DOI:** 10.1001/jamanetworkopen.2024.61038

**Published:** 2025-02-20

**Authors:** Brandy L. Jackson, Vanessa K. Cameron, Tiffany M. Hodgens, Sabrina A. Jamal-Eddine, Vera Kunte, Kathleen Marsala-Cervasio, Lydia Smeltz, Rylee Betchkal, Tess Carichner, Krista Jones, Linda Morrow, Jaclyn Bandell, Katie Pawloski, Anna Valdez, Lisa M. Meeks

**Affiliations:** 1 School of Nursing, Wichita State University, Wichita, Kansas; 2 DocsWithDisabilities Initiative, Access in Nursing Program, Chicago, Illinois; 3 George Washington University School of Nursing, Washington, DC; 4 University of California, Davis, Center for a Diverse Healthcare Workforce; 5 Department of Disability and Human Development, University of Illinois Chicago; 6 School of Nursing, College of Health, Human Science and Nursing, California State University Dominguez Hills; 7 City University of New York, School of Professional Studies, New York, New York; 8 Penn State College of Medicine, Hershey, Pennsylvania; 9 Teachers College, Columbia University, New York, New York; 10 University of Michigan School of Nursing, Ann Arbor; 11 Department of Population Health Nursing Science, University of Illinois Chicago; 12 Sacred Heart University, Davis & Henley College of Nursing, Fairfield, Connecticut; 13 Western Carolina University, School of Nursing, Cullowhee, North Carolina; 14 Florida Gulf Coast University, School of Nursing, Ft Myers; 15 School of Nursing and Health Sciences, Sonoma State University, Rohnert Park, California; 16 Department of Medical Education, University of Illinois College of Medicine, Chicago

## Abstract

This cross-sectional study quantifies disabilities and types of accommodations used among traditional prelicensure nursing students in US Bachelor of Science in Nursing programs.

## Introduction

Medical associations’ commitment to advancing disability-inclusive practices has led to data collection on, and a significant increase in representation of, medical students with disabilities.^1,2^ However, information on disability representation and accommodation use in US nursing programs remains scarce. The lack of data collection on this population impedes the ability to identify barriers, benchmark, and measure progress. To address this gap, we quantified disabilities and types of accommodations used among traditional prelicensure nursing students in US Bachelor of Science in Nursing (BSN) degree programs.

## Methods

This exploratory cross-sectional study, conducted from April 1 through July 30, 2024, used national data from nursing schools to examine disability and accommodation use in traditional prelicensure BSN programs. Participants were recruited through convenience and snowball sampling from social media, American Association of Colleges of Nursing (AACN) listservs, and the AACN newsletter. US traditional prelicensure BSN programs accredited by the Commission on Collegiate Nursing Education were eligible. Postlicensure and accelerated BSN programs were ineligible. The study was exempted and no consent was required by the University of Michigan institutional review board because we collected aggregate data that belong to the institution. We followed the STROBE reporting guideline.

Questionnaires from previous works of the senior author (L.M.M.)^1,2,3^ were adapted from medical to nursing education (eMethods in Supplement 1). The nursing questionnaire collected data on the number of students with disabilities registered with their school’s disability services office by disability category and approved accommodations. Program characteristics, including size, geographic location, private or public designation, and structure of the disability office, were also collected. Schools’ disability resource professionals completed the questionnaire.

Descriptive statistics were used to summarize survey results. To account for heterogeneity between schools, random-effects logistic regression models were used to calculate pooled estimates (weighted by sample size) of disability proportions along with 2-sided 95% CIs. Analyses were conducted using R statistical software, version 4.4.1 (R Project for Statistical Computing).^4^

## Results

Twenty-two schools responded to the social media and listserv call; 19 met criteria for the study and completed the questionnaire. The schools identified 562 of 6416 nursing students with disabilities, representing 8.4% of the total enrollment, with school percentages of nursing students with disabilities ranging from 2% to 21.2% (Table). Psychological disabilities were the most common category reported (224 [3%]), followed by attention-deficit/hyperactivity disorder (ADHD) (141 [2.1%]) and chronic health conditions (98 [1.2%]). Mobility (6 [0.1%]) and sensory (23 [0.4%]) disabilities were less common. School-based testing accommodations were most frequently used (19 [100%]); clinical accommodations were less frequently used (Figure). Nine schools (47.4%) reported using a disability determination structure that included assistance of the disability services office without a liaison.

**Table.** Characteristics of Students With Disabilities and School Profiles in US Traditional Bachelor of Science in Nursing Programs

Characteristic	Value	Weighted proportion, % (95% CI)
**Student characteristics (N = 6416)**		
Students with disabilities, No.	562	8.4 (6.4-10.9)
Type of disability, No.		
Psychological disability (eg, anxiety, depression, bipolar disorders)	224	2.9 (1.9-4.5)
Attention deficit/hyperactivity disorder	141	2.1 (1.7-2.8)
Chronic health condition (eg, lupus, arthritis, chronic back pain)	98	1.2 (0.8-1.9)
Learning disability	50	0.7 (0.4-1.1)
Deaf and hard of hearing	13	0.2 (0.1-0.4)
Other (Tourette syndrome, TBI, stuttering, narcolepsy, ASD)	10	0.1 (0-0.4)
Low vision	10	0.1 (0.1-0.4)
Mobility	6	0.1 (0-0.3)
ASD or autism (from “other”)	5	0.1 (0-0.3)
Acquired or TBI	5	0.1 (0-0.2)
Speech or other communication disability	0	0 (0-1.0)
**School characteristics (N = 19)**		
Public ownership (vs private ownership), No. (%)	15 (78.9)	NA
Disability determination structure, No. (%)		
School of nursing uses the assistance of the disability services office without a liaison	9 (47.4)	NA
School of nursing uses a disability resource professional who works for the health science campus broadly	5 (26.3)	NA
School of nursing uses the assistance of the disability services office	3 (15.8)	NA
School of nursing’s dean of students makes determinations about disability status and accommodations	1 (5.3)	NA
School of nursing uses the assistance of the disability services office, with a specific liaison for nursing	1 (5.3)	NA
BSN program size		
Small (<188 students)	5 (26.3)	NA
Medium (188-400 students)	8 (42.1)	NA
Large (>400 students)	6 (31.6)	NA
Geographic distribution		NA
Northeast	3 (15.8)	NA
South	8 (42.1)	NA
Midwest	7 (36.8)	NA
West	1 (5.3)	NA

Abbreviations: ASD, autism spectrum disorder; BSN, bachelor of science in nursing; NA, not applicable; TBI, traumatic brain injury.

**Figure.**
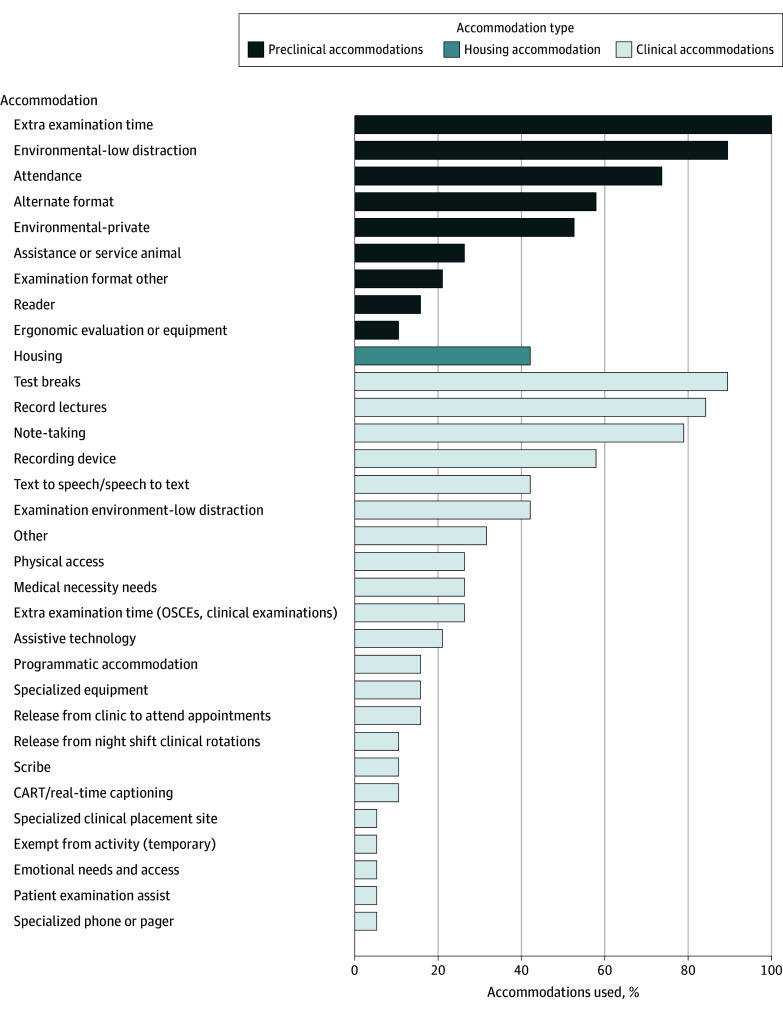
Accommodation Use in US Bachelor of Science in Nursing Programs, by Accommodation Type CART indicates Communication Access Realtime Translation; and OCSEs, Objective Structured Clinical Examinations.

## Discussion

This is the first study, to our knowledge, to evaluate the frequency of nursing students with disabilities in traditional prelicensure BSN programs. We found a proportion estimate of 8.4%, exceeding the prevalence in medical schools (5.9%).^1^ Differences in accommodation provision and disability proportions between schools may stem from variations in admissions practices, disability expertise, or resource allocation. The prominence of psychological disabilities and ADHD suggests these areas should be prioritized in future research, including studies on student performance and efficacy of accommodations. Conversely, the scarcity of nursing students with mobility and sensory disabilities warrants future investigation on barriers to entering and fully participating in the profession.

The use of a convenience sample and resulting small sample size limits generalizability and can amplify the influence of outliers. Although some heterogeneity between schools is expected due to the differences mentioned; a larger, more representative sample would allow these differences to be further explored. However, the accuracy of schools’ reported disability proportions is supported by federally mandated documentation of disability decisions.

This study provides insights into disability, accommodation use, and school-specific differences, including structure of disability determination. Given the valuable contributions of nurses with disabilities to the workforce^5^ and commitments to disability inclusion by nursing associations,^6^ collecting standardized data and prioritizing research on experiences of nursing students with disabilities are essential.

## Notes

Supplement 1. **eMethods.** Questionnaire and Data Requested

Supplement 2.
Data Sharing Statement
