# Supplementary material for: Disability and Accommodation Use in US Bachelor of Science in Nursing Programs
Source: JAMA Netw Open. 2025 Feb 20;8(2):e2461038. doi: 10.1001/jamanetworkopen.2024.61038 (PMC11843349; doi:10.1001/jamanetworkopen.2024.61038)
Supplement: Supplement 1. — eMethods. Questionnaire and Data Requested [file jamanetwopen-e2461038-s001.pdf]

Supplementary Online Content

Jackson BL, Cameron VK, Hodgens TM, et al. Disability and accommodation use in US Bachelor of Science in Nursing programs. *JAMA Netw Open.* 2025;8(2):e2461038. doi:10.1001/jamanetworkopen.2024.61038

eMethods. Questionnaire and Data Requested

This supplementary material has been provided by the authors to give readers additional information about their work.

© 2025 Jackson BL et al. *JAMA Network Open.*

eMethods. Questionnaire and Data Requested

School level data:

Please list the school for which you are submitting information. [open-text response]

Are you accredited by the Commission on Collegiate Nursing Education (CCNE) [yes/no]

Name of the person(s) completing the form.
[open-text response]

Title/Role of the person(s) completing the form.
[open-text response]

Email of the person(s) completing the form.
[open-text response]

What is the total size (in all years) of your **Pre-licensure BSN Program?**
[open-text response]

Does your program employ any staff or faculty members with disabilities?
Yes/ No/ Unknown

Is your program in a public or private institution?
Public/Private

Please select the option that most accurately describes the structure for disability determination at your nursing school.

- The School of Nursing **employs a nursing-specific disability resource professional** who reviews requests for accommodation.
- The School of Nursing utilizes a **disability resource professional who works for the health science campus** broadly.
- The School of Nursing utilizes the assistance of **the disability services office**, **with a specific liaison for nursing.**
- The School of Nursing utilizes the assistance of the **disability services office without a liaison.**
- The School of Nursing utilizes an **internal committee of faculty and administrators to make determinations about disability status and accommodations.**
- The **School of Nursing's dean of students makes determinations** about disability status and accommodations.

Disability Office Data:

In case we have questions about the data please let us know who to contact:

Name of the person(s) completing the form.
[open-text response]

Title/Role of the person(s) completing the form.
[open-text response]

© 2025 Jackson BL et al. *JAMA Network Open.*

Email of the person(s) completing the form.
[open-text response]

Please provide the total aggregate number of Pre-licensure BSN Students that are currently registered with the Disability Services regardless of whether they currently receive accommodation.
[open text response]

Of the total registered traditional prelicensure BSN Students with disability services, please provide the aggregate number registered under each of the following disability categories using primary disability type.

Acquired/Traumatic Brain Injury:	[open-text response]
Attention Deficit/Hyperactivity Disorder:	[open-text response]
Chronic health condition (e.g. lupus, arthritis, chronic back pain):	[open-text response]
Deaf and Hard of Hearing:	[open-text response]
Learning Disability:	[open-text response]
Mobility:	[open-text response]
Psychological Disability:	[open-text response]
Speech/Other Communication Disability:	[open-text response]
Vision - Low Vision:	[open-text response]
Other (please specify below) (Tourette's, TBI, Stuttering, Narcolepsy, Autism Spectrum Disorder).	[open-text response]
Please enter one line for each additional disability service and include the number of students in this category.	[open-text response]

Of all the pre-licensure BSN students registered with disability services, please provide the aggregate number granted each of the following accommodations for the didactic portion of their education? Total number of responses may be greater than the total number of students registered with disability services, given that most students have more than one accommodation.

Assistance or Service Animal:	[open-text response]

© 2025 Jackson BL et al. *JAMA Network Open.*

Alternate Format (textbook conversion, other):	[open-text response]
Attendance:	[open-text response]
Environmental - Low Distraction:	[open-text response]
Environmental - Private:	[open-text response]
Ergonomic Evaluation or Equipment:	[open-text response]
Extra Exam Time:	[open-text response]
Exam Format Other (please enter number then describe below):	[open-text response]
Exam Format Other (please describe):	[open-text response]
Housing (Single Room, Release, Parking):	[open-text response]
Recording Device:	[open-text response]
Notetaking:	[open-text response]
Programmatic Accommodation (e.g., decelerated curriculum) Please enter number then describe below:	[open-text response]
Programmatic Accommodation (please describe):	[open-text response]
Record Lectures:	[open-text response]
Test Breaks:	[open-text response]
Text to Speech/Speech to Text (Screen reader, Dragon):	[open-text response]

Of all pre-licensure BSN students registered with disability services, please provide the aggregate number granted each of the following accommodations for the clinical portion of their education? Total number of responses may be greater than the total number of students registered with disability services, given that most students have more than one accommodation.

© 2025 Jackson BL et al. *JAMA Network Open.*

Ability to perform procedural skill or clinical competency in simulation lab:	[open-text response]
Assistive Technology (e.g., Specialized stethoscope otoscope, other devices):	[open-text response]
CART/Real-Time Captioning:	[open-text response]
Decelerated Clinical Rotation:	[open-text response]
Extra Exam Time (e.g., OSCEs):	[open-text response]
Exam Reduced Distraction Environment:	[open-text response]
Intermediary or assistant to facilitate patient exam:	[open-text response]
Release from clinic to attend appointments:	[open-text response]
Release from night shift clinical rotations:	[open-text response]
Scribe:	[open-text response]
Sign Language Interpreter or Cued Speech:	[open-text response]
Specialized Clinical Placement Site:	[open-text response]
Specialized Phone or Pager:	[open-text response]
Specialized Equipment (amplified stethoscope, computer, WOW station):	[open-text response]
Exempt from activity for limited period due to temporary injury:	[open-text response]
Other (please enter total number and then enter details below):	[open-text response]

© 2025 Jackson BL et al. *JAMA Network Open.*
