# Supplementary material for: Disability and Accommodation Use in US Bachelor of Science in Nursing Programs
Source: JAMA Netw Open. 2025 Feb 20;8(2):e2461038. doi: 10.1001/jamanetworkopen.2024.61038 (PMC11843349; doi:10.1001/jamanetworkopen.2024.61038)
Supplement: Supplement 2. — Data Sharing Statement [file jamanetwopen-e2461038-s002.pdf]

Data Sharing Statement

Jackson. Disability and Accommodation Use in US Bachelor of Science in Nursing Programs. *JAMA Netw Open*. Published February 20, 2025. doi:10.1001/jamanetworkopen.2024.61038

Data
Data available: No

Additional Information
Explanation for why data not available: our DLA with schools does not allow for this
